# Conduction Band Energy‐Level Engineering for Improving Open‐Circuit Voltage in Antimony Selenide Nanorod Array Solar Cells

**DOI:** 10.1002/advs.202100868

**Published:** 2021-06-10

**Authors:** Tao Liu, Xiaoyang Liang, Yufan Liu, Xiaoli Li, Shufang Wang, Yaohua Mai, Zhiqiang Li

**Affiliations:** ^1^ National‐Local Joint Engineering Laboratory of New Energy Photoelectric Devices College of Physics Science and Technology Hebei University Baoding 071002 China; ^2^ Institute of New Energy Technology College of Information Science and Technology Jinan University Guangzhou 510632 China

**Keywords:** gradient band structure, heterojunction interface, In_2_S_3_‐CdS composite buffers, Sb_2_Se_3_ nanorod arrays, solar cells

## Abstract

Antimony selenide (Sb_2_Se_3_) nanorod arrays along the [001] orientation are known to transfer photogenerated carriers rapidly due to the strongly anisotropic one‐dimensional crystal structure. With advanced light‐trapping structures, the Sb_2_Se_3_ nanorod array‐based solar cells have excellent broad spectral response properties, and higher short‐circuit current density than the conventional planar structured thin film solar cells. However, the interface engineering for the Sb_2_Se_3_ nanorod array‐based solar cell is more crucial to increase the performance, because it is challenging to coat a compact buffer layer with perfect coverage to form a uniform heterojunction interface due to its large surface area and length–diameter ratio. In this work, an intermeshing In_2_S_3_ nanosheet‐CdS composite as the buffer layer, compactly coating on the Sb_2_Se_3_ nanorod surface is constructed. The application of In_2_S_3_‐CdS composite buffers build a gradient conduction band energy configuration in the Sb_2_Se_3_/buffer heterojunction interface, which reduces the interface recombination and enhances the transfer and collection of photogenerated electrons. The energy‐level regulation minimizes the open‐circuit voltage deficit at the interfaces of buffer/Sb_2_Se_3_ and buffer/ZnO layers in the Sb_2_Se_3_ solar cells. Consequently, the Sb_2_Se_3_ nanorod array solar cell based on In_2_S_3_‐CdS composite buffers achieves an efficiency of as high as 9.19% with a *V*_OC_ of 461 mV.

## Graphical abstract

An intermeshing In_2_S_3_ nanosheet‐CdS composite buffer layer is constructed and applied in Sb_2_Se_3_ nanorod array solar cells, which build a gradient conduction band energy configuration in the Sb_2_Se_3_/buffer heterojunction interface. The champion cell based on In_2_S_3_‐CdS composite buffers achieves a PCE of 9.19% with an *V*_OC_ of as high as 461 mV.

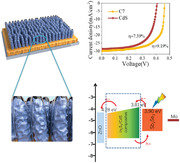

## 1 Introduction

Photovoltaic (PV) technologies that directly convert solar radiation into electricity, offer a green and renewable energy to meet the growing demand in the developed world. Among thin film solar cells, great successes have been achieved in copper indium gallium selenide (CIGS), cadmium telluride (CdTe), and perovskites with certified power‐conversion efficiency (PCE) over than 22%.^[^ ^1^, ^2^, ^3^, ^4^ ^]^ However, the toxicity, natural instability, and limited abundance of some of their constituent elements might be the obstacles for large‐scale application.^[^ ^5^, ^6^ ^]^ Thus, alternative earth‐abundant light‐absorbing materials have been investigated for high efficiency, eco‐friendly, and stable solar cells.

Antimony selenide (Sb_2_Se_3_) has recently emerged as a promising PV absorber material and been receiving more and more research interest due to its favorable material and optoelectronic properties.^[^ ^7^, ^8^, ^9^ ^]^ It exhibits proper bandgap of 1.1–1.3 eV, high absorption coefficient of 10^5^ cm^−1^ in the visible region, excellent carrier mobility, and good device stability.^[^ ^10^, ^11^ ^]^ Moreover, Sb_2_Se_3_ is environmentally friendly, and is not in the list of highly materials by US, EU, or Chinese regulation authorities. The abundant of elements Sb and Se in the earth's crust is 0.2 and 0.05 ppm, respectively.^[^ ^7^, ^11^ ^]^ Consequently, this makes Sb_2_Se_3_ a very promising absorber material for PV application.^[^ ^12^, ^13^, ^14^ ^]^

The most attractive merit of Sb_2_Se_3_ is the one‐dimensional (1D) nanoribbon grain structure comprising of covalently bonded (Sb_4_Se_6_)*_n_* ribbons held together via Van der Waals forces.^[^ ^15^, ^16^ ^]^ This unique 1D crystal structure leads to not only the bond anisotropy but also the strongly anisotropy in optical, electrical, and defect properties. For instance, the hole mobility along ribbon (c‐direction) is nearly four times than that cross the ribbons (b‐direction).^[^ ^10^, ^17^ ^]^ Thus, control of the crystal orientation of Sb_2_Se_3_ grains is the very crucial to the quality of absorber layer and also the device performance of solar cells.^[^ ^18^, ^19^ ^]^ Tang's group reported that the strong correction between device performance and the orientation of Sb_2_Se_3_ absorber layer.^[^ ^15^ ^]^ The detailed analysis revealed that the carrier transport in the [221]‐oriented grain (traveling within the covalently bonded (Sb_4_Se_6_)*_n_* ribbons) was much easier than in the [120]‐oriented grains (charge hopping between ribbons held together by Van der Waals force). They further investigated the effect of ZnO substrate orientation on the orientation and quality of the top Sb_2_Se_3_ layer.^[^ ^10^ ^]^ As a result, highly preferred [221] orientation Sb_2_Se_3_ absorber was obtained, and the corresponding superstrate ZnO/Sb_2_Se_3_ thin film solar cell demonstrated a high conversion efficiency and good stability under the test condition of International Electrochemical Commission 61 646 protocol.^[^ ^10^ ^]^ Up to now, most reported Sb_2_Se_3_ solar cells are based on [221]‐oriented Sb_2_Se_3_ absorber layer with excellent carrier transport property.^[^ ^20^, ^21^, ^22^, ^23^, ^24^ ^]^

Recently, we reported the construction of photovoltaic devices based on Sb_2_Se_3_ nanorod arrays along the [001] direction, where the single crystal Sb_2_Se_3_ nanorod consists of titled (Sb_4_Se_6_)*_n_* ribbons stacked vertically on the substrate.^[^ ^25^ ^]^ The [001]‐oriented Sb_2_Se_3_ grain allows the carrier transport only in the covalent‐bonded ribbons and is beneficial for device performance improvement. In addition, the growth of 1D [001]‐oriented Sb_2_Se_3_ nanorod is thermodynamically favorable, and results in no dangling bonds at their grain boundaries (GBs) due to the low formation energies. The potential issue of carrier recombination loss at the GBs could be alleviated for the 1D Sb_2_Se_3_ nanorod‐based solar cells, whereas the recombination through dangling bonds at the GBs is the main factor limiting the device performance for traditional 3D ploy‐crystalline thin film solar cells, such as CuInGaSe_2_, CuInS_2_, CdTe, CZTS, etc.^[^ ^26^ ^]^ From this view, the interface recombination, especially occurs at the terminals of the single‐crystal Sb_2_Se_3_ nanorod, become dominant for the performance improvement of Sb_2_Se_3_‐based solar cells. The Sb_2_Se_3_ nanorod array‐based solar cells suffer from higher interface defect density and carrier recombination and thus have more serious open‐circuit voltage deficit than the Sb_2_Se_3_ thin film configuration devices.^[^ ^21^, ^27^, ^28^, ^29^, ^30^ ^]^ The element interdiffusion at the junction interface is another interesting feature of Sb_2_Se_3_/CdS heterojunction solar cells.^[^ ^31^, ^32^ ^]^ Williams et al. revealed that an atom scale interfacial layer can unavoidably form at the Sb_2_Se_3_/CdS interface due to the rapid interdiffusion of Sb and Se from Sb_2_Se_3_, which would increase the interfacial recombination and lead to low device efficiencies.^[^ ^33^ ^]^ An ultrathin titanium oxide (TiO_2_) layer deposited by atomic layer deposition (ALD) has been employed to passivate the Sb_2_Se_3_ nanorod array‐based heterojunction interface, and an power conversion efficiency (PCE) as high as 9.2% was achieved.^[^ ^25^ ^]^ The interface engineering was a crucial factor for high efficiency Sb_2_Se_3_ solar cells. Vacuum thermal evaporated buffer layer was also employed in the substrate Sb_2_Se_3_ devices with following chemical etching and annealing procedure.^[^ ^34^ ^]^

In this work, we propose a solution processed In_2_S_3_‐CdS composite buffer layer application in Sb_2_Se_3_ nanorod array solar cells. This fabrication procedure is vacuum‐free and low‐cost in comparison with ALD‐TiO_2_ technique. Meanwhile, the band alignment structure of the heterojunction is another crucial factor that relating to the interface recombination behavior and determining the efficiencies of thin film solar cells. A cliff‐like band offset is observed at the Sb_2_Se_3_/CdS interface, which would increase the interface recombination and thus decrease the device *V*_OC_.^[^ ^35^ ^]^ The band structure of In_2_S_3_‐CdS composite buffer could be controlled by regulating the deposition of CdS layer. The composite buffer reduces the recombination paths at the heterojunction interface, leading to an increase in recombination activation energy *E*_a_ and *V*_OC_. Moreover, a gradient conduction band maximum is formed for the In_2_S_3_‐CdS composite layer, which is favorable for the transfer and collection of photogenerated electrons. as a result, a PCE of 9.19% is achieved for device based the optimized composite buffer with *V*_OC_ of 461 mV, *J*_SC_ of 29.92 mA cm^−2^ and FF of 66.67%, while the PCE of the standard device with single CdS buffer is 7.39%.

## 2 Results and Discussion

**Figure 1:**
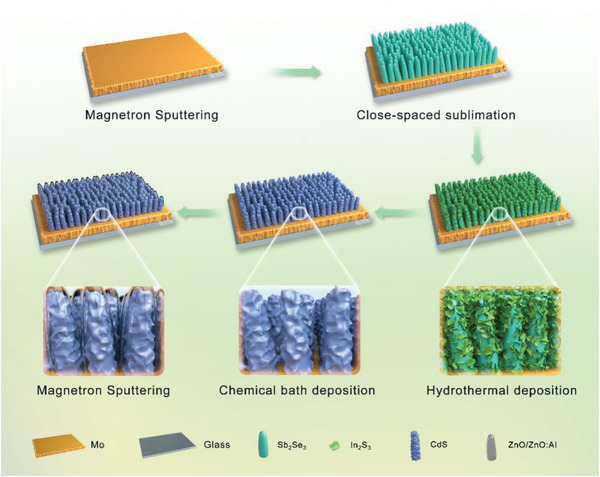
Illustration of deposition processes of the Sb_2_Se_3_ nanorod array solar cells with In_2_S_3_‐CdS composite buffers.

As illustrated in **Figure** **1**, in this work, the Sb_2_Se_3_ nanorod arrays (NRAs) were prepared by close spaced sublimation (CSS) technique by subliming the Sb_2_Se_3_ powder source onto the surface selenized Mo‐coated glass substrate. The sublimation process continues for tens of seconds and the thickness of Sb_2_Se_3_ NRAs is about 1000–1200 nm. Afterward, the Sb_2_Se_3_ NRAs were first coated by solution‐processed indium sulfide (In_2_S_3_) nanosheet layer, and followed by deposition of cadmium sulfide coating to get the In_2_S_3_‐CdS composite buffers. We first check the morphologies and compositions of the In_2_S_3_‐CdS composite layers, and the single In_2_S_3_ nanosheet layer and single CdS layer were also included for comparison. In this work, the In_2_S_3_ nanosheet layer and CdS layer were prepared by hydrothermal deposition and chemical bath deposition (CBD), respectively. The composite layers were controlled by tuning the CdS deposition time between 5 and 11 min, while keeping the In_2_S_3_ deposition condition was same for all samples. For description clarity, we denoted the sample coated with hydrothermal In_2_S_3_ and CdS buffer for 5 min as C5, and CBD CdS layer for 7, 9, and 11 min as C7, C9, and C11, respectively.

**Figure 2:**
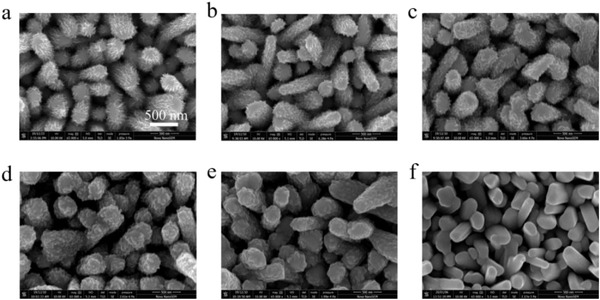
Top‐view SEM micrographs of the Sb_2_Se_3_ nanorod arrays with different buffer layers. a) Single In_2_S_3_, b–e) In_2_S_3_‐CdS composites b) C5; c) C7; d) C9; e) C11, and f) single CdS.

**Figure** **2** shows the top‐view scanning electron microscope (SEM) images of Sb_2_Se_3_ NRAs coated with In_2_S_3_, In_2_S_3_‐CdS composites (C5, C7, C9, C11), and single CdS buffer layers. Obvious nanosheet‐like In_2_S_3_ films could be observed on the surface of Sb_2_Se_3_ nanorods after the hydrothermal deposition of In_2_S_3_ for 100 min (Figure 2a; and Figure S2a, Supporting Information), resulting in a dendritic Sb_2_Se_3_/In_2_S_3_ core/shell heterojunction. The X‐ray diffraction (XRD) pattern (Figure S1, Supporting Information) indicates that the peaks can be indexed to pure cubic *β*‐In_2_S_3_ phase (JCPDS 65‐0459). After deposition of 5 min CdS, the dendritic features of the NRAs become weakened but remain loose nanorod surface (C5, Figure 2b). With further increase in the CBD reaction time to 9 (C9) and 11 min (C11), the dendritic NRAs disappears and dense and slightly rough surface formed on the Sb_2_Se_3_ nanorod (Figure 2e). In contrast, for the NRAs coated with single CBD CdS layer, a fully conformal and intact coating of CBD processed CdS layer on the Sb_2_Se_3_ NRAs are obtained, and maintains the smooth nanorod surface, as shown in Figure 2f.

This strongly difference in morphology could be ascribed to the different mechanism for the growth of CdS and In_2_S_3_. The formation of In_2_S_3_ nanoflakes is based on the slow release of In^3+^ and S^2−^ ions in an acidic solution, and the following nucleation and growth stages on the Sb_2_Se_3_ nanorod surface when the ionic product exceeds the solubility product, which is a typical ion‐by‐ion mechanism.^[^ ^36^, ^37^ ^]^ Ions, including In^3+^, C_6_H_7_O_7_^−^(L^−^), S^2−^, H^+^ and other ions, were formed in the reaction aqueous solution by ionization and hydrolysis action from the precursors (indium chloride, citric acid and TAA) in the aqueous solution. *β*‐In_2_S_3_ can be formed under a low concentration of S^2−^ due to its low solubility of 5.7 × 10^−74^.^[^ ^36^ ^]^ Citrate ion (C_6_H_7_O_7_^−^) combines with some of In^3+^ to reduce the concentration of free In^3+^ in an acidic solution (pH = 2). In_2_S_3_ nuclei can be formed by directly combination of In^3+^ and S^2−^ under a low concentration. On the other hand, citrate ion could also be combined with some of In^3+^ to form the indium‐citrate complex ion In(C_6_H_7_O_7_^−^)_3_, and play role in controlling the concentration of free In^3+^ in the solution. This allows the formation of In_2_S_3_ nuclei on the Sb_2_Se_3_ nanorod surface. Then the nuclei on the nanorod surface grow self‐assembled and gradually to form nanoflakes due to the difference in surface energy of *β*‐In_2_S_3_.^[^ ^36^, ^38^ ^]^

In contrast, the formation of CdS layer is normally following a complex‐decomposition cluster mechanism, which is based on the formation of an intermediate complex with the anion‐forming reagent instead of reacting directing with a free anion.^[^ ^37^, ^39^, ^40^ ^]^ The growth of CdS occurs in an alkaline solution consisting of ammonium hydroxide providing ammonia in the solution. The cadmium ions (Cd^2+^), generated from the cadmium salt, complex with ammonia to form amino‐cadmium complex ion Cd(NH_3_)_4_^2+^, controlling the concentration of free cadmium ions. Then, Cd(NH_3_)_4_^2+^ complex ion reacts with hydroxide ions to form the adsorbed dihydroxo‐diammion‐cadmium complex [Cd(OH)_2_(NH_3_)_2_]_ads_ on the In_2_S_3_ flake or Sb_2_Se_3_ nanorod surface. This complex then reacts with thiourea to form the metastable complex [Cd(OH)_2_(NH_3_)_2_SC(NH_2_)_2_]_ads_. The final CdS was formed through decomposition of the metastable complex. In this stage, the formation of CdS is more likely an in situ and conformal growth process. Furthermore, as the reaction time prolonging, the CdS become bigger nanoparticles gradually and closer together to form continuous films, and the growth rate is high in this stage. This could explain that the huge difference in morphology for different deposition time of CdS, as shown in Figure 2. Insufficient deposition time of CdS (7 and 5 min) on the rough Sb_2_Se_3_/In_2_S_3_ nanorod surface maintains the morphology of In_2_S_3_ flake (Figure 2b–c). However, when the reaction time prolongs to 11 min or more, CdS could fill the space between the In_2_S_3_ flakes, and results in a smooth surface.

**Figure 3:**
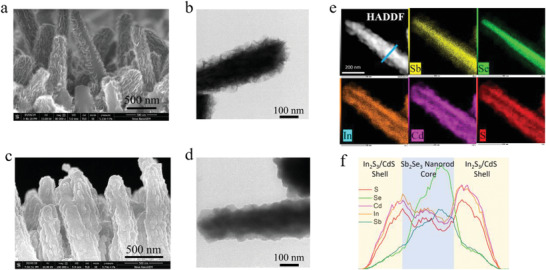
a) SEM of the Sb_2_Se_3_/In_2_S_3_ core/shell nanorod arrays. b) TEM image of one Sb_2_Se_3_/In_2_S_3_ core/shell nanorod. c) SEM image of Sb_2_Se_3_/In_2_S_3_‐CdS core/shell nanorod arrays. d) TEM image of one Sb_2_Se_3_/In_2_S_3_‐CdS core/shell nanorod. e) HAADF‐STEM images and EDS element mapping of the Sb_2_Se_3_/In_2_S_3_‐CdS core/shell nanorod. f) Line‐scan of the Sb_2_Se_3_/In_2_S_3_‐CdS core/shell nanorod.

**Figure** **3**; and Figure S3 (Supporting Information) display the difference in morphology and composition in detail between the Sb_2_Se_3_ NRAs coated with In_2_S_3_ nanoflake films and with In_2_S_3_‐CdS composite (C7) films. As shown, the surface of Sb_2_Se_3_ nanorod is covered by a shell of nanoflake film, like prickly tree branches. High‐magnification TEM image in Figure 3b shows the In_2_S_3_ nanoflakes are tightly attached to the Sb_2_Se_3_ nanorod surface, forming a core–shell nanorod structure. In contrast, after several minutes of deposition of CdS, the prickly‐like nanorod surface became slightly smooth, as shown in Figure 3c,d. High‐angel annular dark‐field scanning transmission electron microscope (HAADF‐STEM) equipped with energy dispersive spectroscopy (EDX) measurement was performed to characterize the composition of this core/shell nanorod structures. Single nanorod was chosen to analyze the Sb, Se, In, Cd, and S element distribution for the core/shell heterostructure and the results are shown in Figure 3f. it exhibits that the Sb and Se elements were distributed in the core, and the copresence of Cd, In, and S elements in the shell of the nanorod heterostructure. On the contrary, only In and S elements are observed in the shell of Sb_2_Se_3_/In_2_S_3_ heterojunction nanorod (Figure S3, Supporting Information). A line scan (Figure 3f) further supported this claim. Both shoulders of In, Cd, and S could be observed in the line, corresponding to the In_2_S_3_‐CdS nanocomposite shell. The element distribution mapping and EDX line scan results confirm that the Sb_2_Se_3_ nanorod and In_2_S_3_‐CdS composite buffer layer core/shell NRAs are successfully constructed on the surface via hydrothermal and following CBD method.

**Figure 4:**
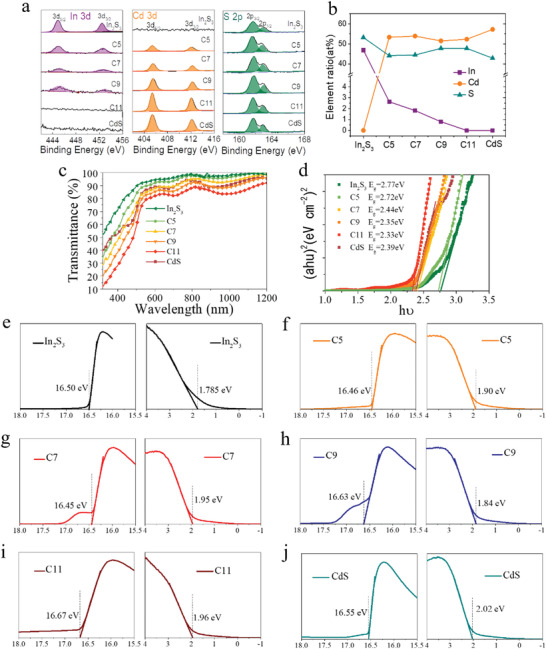
Surface chemical analysis. a) XPS peak of Cd 3d, In 3d, and S 2p, respectively, obtained from the surface of Sb_2_Se_3_ NRAs coated with CdS, In_2_S_3_‐CdS composites, and In_2_S_3_ buffer layers. b) The composition element ratios of the buffer layers. c) The optical transmittance spectra, and d) Tauc plots of the different buffer layers. e–j) Surface UPS spectra of these buffer layers, e) In_2_S_3_, f) C5, g) C7, h) C9, i) C11, and j) CdS.

X‐ray photoelectron spectroscopy (XPS) characterization was performed to check the chemical states of the Sb_2_Se_3_ NRAs coated with different buffers (In_2_S_3_, In_2_S_3_‐CdS composites, and CdS). Curve fitting was further performed to determine the surface composition and chemical state of Cd, In, and S with different deposition process, as shown in **Figure** **4**a. In S 2p spectra, the peaks at 161.5 and 162.7 eV, which corresponds to S 2p_3/2_ and S 2p_1/2,_ respectively, were detected from all the samples. In the Cd 3d core level XPS spectra, the peaks at 405.2 and 411.9 eV, corresponding to Cd 3d_5/2_ and Cd 3d_3/2_ of CdS, respectively, were detected from all the samples but the NRAs with only In_2_S_3_ coating. In the In 3d spectra, the peaks associating with In 3d_5/2_ and In 3d_3/2_ of In_2_S_3_, were detected from the In_2_S_3_, C5, C7, and C9 samples. The peak intensity decreases with the increase of deposition of CBD CdS layer, suggesting that the growth of CdS not only between the space of In_2_S_3_ flakes but also covered on flake surface. Moreover, no obvious peaks corresponding to In 3d are observed for the C11 sample, hinting that the good coverage of CdS layer on the In_2_S_3_ nanoflake films.

The UV–vis and ultraviolet photoelectron spectroscopy (UPS) was employed to reveal the band structure of the composite buffer layers. Figure 4c shows the optical transmittance spectra of the layers. The corresponding effective bandgaps (*E*_g_) can be obtained based on the Tauc equation, and the calculated results are shown in Figure 4d. The *E*_g_ is 2.77, 2.72, 2.44, 2.35, 2.33, and 2.39 eV for single In_2_S_3_ nanoflake film, C5, C7, C9, C11, and single CdS film, respectively. At first, we character the surface energy level of the films. The Fermi level, valence band maximum (VBM) of the sample surfaces were obtained by fitting the cut‐off binding energy and long‐tails of the UPS spectra (Figure 4e–j). The conduction band maximum (CBM) was determined from the *E*_g_ (Figure 4d) and VBM position. The CBM level is 3.74, 3.94, 4.28, 4.08, 4.18, and 4.30 eV for the single In_2_S_3_ nanoflake film, C5, C7, C9, C11, and single CdS film, respectively. Compared to the CdS layer, a slightly upshift of the CBM is observed for the In_2_S_3_‐CdS composite layers. It suggests that the band structure (bandgap, CBM, VBM) of the combination of In_2_S_3_ and CdS is affected by the element‐content and therefore depend on the CdS deposition time as the In_2_S_3_ layer has the same deposition condition for each sample.

In fact, the In_2_S_3_‐CdS composite films (C5, C7, C9, or C11) is not stacked layer by layer, which more likely to be a mixture of In_2_S_3_ and CdS. The Sb_2_Se_3_ nanorod is first capped by In_2_S_3_ nanoflake films with a poor coverage due to the its ion‐by‐ion growth mechanism. Then, the following CdS layer caped both In_2_S_3_ nanoflake and the rest of Sb_2_Se_3_ nanorod surface to form a mixed buffer layer. As the CdS deposition time was longer than 9 min, the properties of CdS is dominant and the properties of In_2_S_3_‐CdS composite buffers vary slightly with the CdS thickness. The surface CBM of the In_2_S_3_‐CdS composites could be tuned by regulating the deposition time of CBD‐CdS. It shows a trend of first decreasing, then increasing and then reducing with decreasing the CdS deposition time, suggesting that the interface between In_2_S_3_‐CdS composite buffers and Sb_2_Se_3_ absorber has a tendency for a spike‐like conduction band offset (CBO).

**Figure 5:**
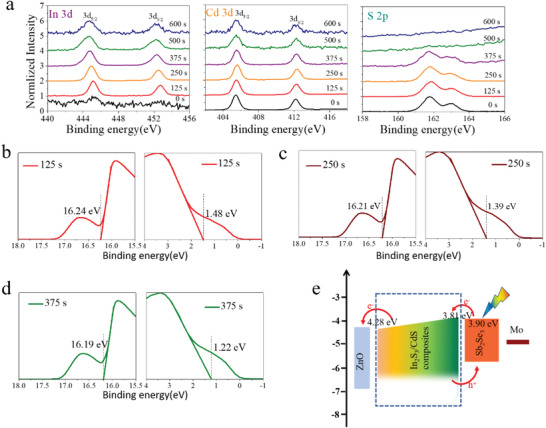
In‐depth XPS and UPS analysis of In_2_S_3_‐CdS composite layer (C7). a) In 3d, Cd 3d, and S 2p XPS spectra of the C7 with different etching time (0, 125, 250, 375, 500, and 600 s). b–d) UPS spectra of C7 at different depths during sputtering. b) 125 s, c, 250 s, and d) 375 s. e) Schematic diagram of band structures and carrier transport of the Sb_2_Se_3_ solar cell with an In_2_S_3_/CdS composite layer.

Due to the special structure of In_2_S_3_ nanoflake and CdS nanocomposites, we further characterize in detail the in‐depth composition and band structures of the In_2_S_3_‐CdS composite layer (C7) by performing the in‐depth XPS and UPS measurement. **Figure** **5**a illustrates the XPS depth profiling spectra of C7. Spectra of the binding energy of In 3d, Cd 3d, and S 2p core levels are presented. On the sample surface, Cd and S are clearly detected in the first few nanometers of the C7 sample surface as shown in the Cd 3d and S 2p spectra, whereas no obvious In is detected. After etching for 125 s (the reference etching rate is 0.60 nm s^−1^ for Ta_2_O_5_), In is clearly detected at the near‐surface region, and Cd and S peaks remain nearly uncharged, indicating that copresence of In_2_S_3_ and CdS in the near‐surface region. The In, Cd, and S peaks keep nearly the same intensity as the etching time increases to 375 s. Furthermore, the In and Cd peaks decrease, and the S peak even disappears once the etching time increase to 500 s. This hints that the rear surface of C7 was reached for the etching time between 375 and 500 s. In the following UPS depth profile analysis, the spectra of the C7 sample with etching for 375 s could reveal the band structure of C7 rear surface. The corresponding UPS cutoff spectra of C7 for different etching time (125, 250, and 375 s) are shown in Figure 5b–d, respectively. As expected, the band structure shows an in‐depth nonuniform behavior. The calculated VBMs and work functions are 1.48 and 16.24 eV for C7 with 125 s etching, 1.39 and 16.21 eV for 250 s, and 1.22 and 16.19 eV for 375 s, respectively. Thus, the CBM values are 4.02, 3.96, and 3.81 eV for C7 with etching time of 125, 250, and 375 s, respectively. These measurement results suggest that the In_2_S_3_‐CdS composites layer (C7) has a gradual CBM structure (4.28 eV for the surface and 3.81 eV for the rear surface). The CBM of rear surface of C7 layer is slightly higher than that of the Sb_2_Se_3_ absorber (3.90 eV, Figure S4, Supporting Information).

**Figure 6:**
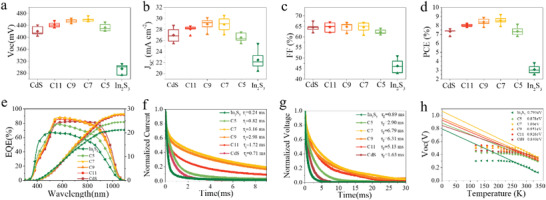
a–d) Statistics of device parameters for the Sb_2_Se_3_ solar cells with different buffers. a) *V*_OC_, b) *J*_SC_, c) FF, and d) PCE. e) EQE spectra of Sb_2_Se_3_ solar cells with different buffers. f,g) Normalized log scale current decay f) and photovoltage decay g) measurements for the Sb_2_Se_3_ solar cells. h) Temperature dependent *V*_OC_.

To clarify the relationship between the different types of buffer layers and the photovoltaic properties of the Sb_2_Se_3_ solar cells, we fabricated the Sb_2_Se_3_ solar cells in a typical substrate configuration of Mo/MoSe_2_/Sb_2_Se_3_/buffer (CdS, In_2_S_3_‐CdS, In_2_S_3_)/ZnO/Al:doped ZnO. **Figure** **6**a–d and **Table** **1** show the statistic device parameters with different buffers. The typical CdS single buffer‐based Sb_2_Se_3_ solar cell exhibits an open‐circuit voltage (*V*_OC_) of 405 mV, a short‐circuit current density (*J*_SC_) of 28.70 mA cm^−2^, a fill factor (FF) of 63.60%, and a power conversion efficiency (PCE) of 7.39%. Meanwhile, the typical In_2_S_3_ single buffer‐based cell shows a PCE of 3.41% from the declining V_OC_, *J*_SC_, and FF. Notably, it is clear that all devices with In_2_S_3_‐CdS composite buffers (C5, C7, C9, and C11) demonstrate remarkable enhancement in *V*_OC_, *J*_SC_, and also PCE than the devices with CdS or In_2_S_3_ single buffer. The highest PCE of 9.19% is achieved for device with C7 buffer with *V*_OC_ of 461 mV, *J*_SC_ of 29.92 mA cm^−2^, and FF of 66.67%. It is worth noting that *V*_OC_ was promoted from 405 to 461 mV after the application and optimization of In_2_S_3_‐CdS composite buffers, which will be discussed later. On the other hand, the *J*_SC_ of solar cells with composite buffers exhibit slightly higher values than the device with CdS single buffer, and much higher than the device with single In_2_S_3_ buffer.

**Table 1:** Champion and average device parameters for solar cells based on different buffers

Buffer	*V*_OC_ [mV]	*J*_SC_ [mA cm^−2^]	FF [%]	PCE [%]
**In_2_S_3_**	293 ± 15 (302)	22.1 ± 1.0 (23.2)	46.3 ± 3.4 (48.60)	3.01 ± 0.37 (3.41)
**C5**	433 ± 12 (433)	26.50 ± 0.8 (27.40)	62.3 ± 1.1 (63.30)	7.15 ± 0.28 (7.51)
**C7**	460 ± 6 (461)	28.9 ± 1.20 (29.92)	64.6 ± 2.6 (66.67)	8.55 ± 0.42 (9.19)
**C9**	455 ± 6 (455)	28.9 ± 1.0 (29.58)	64.8 ± 1.9 (65.80)	8.38 ± 0.37 (8.85)
**C11**	442 ± 8 (435)	28.0 ± 0.6 (28.27)	64.7 ± 2.0 (66.70)	8.00 ± 0.14 (8.20)
**CdS**	420 ± 14 (405)	26.9 ± 1.3 (28.70)	64.5 ± 1.7 (63.60)	7.32 ± 0.24 (7.39)

The external quantum efficiencies (EQEs) of champion cells from each group are displayed in Figure 6e. In the short wavelength range (350–600 nm), the EQE shows an obvious CdS thickness‐dependent behavior, where the EQE increase as the CdS deposition time decreases. The cell with In_2_S_3_ single buffer has a better photospectral response in this short‐wavelength range. This could be attributed to its thinner thickness than the In_2_S_3_‐CdS buffers and bigger bandgap than the CdS layer, which allow more photons pass through and be absorbed by the Sb_2_Se_3_. This could be ascribed to the narrow bandgap of CdS (2.39 eV) thin films, and the photons was absorbed by the CdS buffer before it arrived the Sb_2_Se_3_ layer without contribution much to the photocurrent due to its high doping density.^[^ ^41^ ^]^ In the medium wavelength range (500–750 nm), the EQE was determined the Sb_2_Se_3_ absorber and absorber/buffer heterojunction interface quality. In comparison with CdS/Sb_2_Se_3_ interface, the coverage for In_2_S_3_ nanosheet on the Sb_2_Se_3_ rod surface is poor. The exposed Sb_2_Se_3_ surface is covered by the following CdS, and the coverage is improved after the formation of In_2_S_3_‐CdS composite buffers. Hence, devices with composite buffers (C7, C9, C11) exhibit higher values than the reference CdS device, suggesting the variation in the heterojunction. In the longer wavelength range (750–1000 nm), the EQE values for devices with composite buffers show slight decrease. It could be ascribed to the incomplete collection of photogenerated carriers in the absorber, and the biased EQE measurement at −0.5 V (Figure S6, Supporting Information) show enhanced values in the longer wavelength range.

*J*_SC_‐decay and *V*_OC_‐decay were conducted to explore the photogenerated carrier dynamics in the solar cells.^[^ ^29^, ^42^ ^]^ The charge transport lifetime (*τ_t_*) could be derived from the *J*_SC_‐decay curves, while the *V*_OC_‐decay curves are corrected to the carrier recombination rates under the open‐circuit conditions in the full solar cells. Figure 6g demonstrates the *V*_OC_‐decay curves of the Sb_2_Se_3_ nanorod solar cells with various buffers. The curves for the composite buffer‐based solar cells exhibit much slower decays, in comparison with the reference CdS buffer‐based device. The charge recombination lifetime (*τ_r_*) derived from the *V*_OC_‐decay curves was calculated to be 6.79 and 1.63 ms for C7 and CdS device, respectively. This suggested that the carrier recombination was dramatically prohibited by involving the In_2_S_3_‐CdS composite buffer in the device.

Temperature‐dependent current density voltage (*J–V–T)* measurement was further carried out to investigate the mechanism of carrier transport at the heterojunction interface, as shown in Figure 6h. The activation energy of the main recombination paths can be obtained from the *V*_OC_ versus temperature plot, and the relationship can be described by the following equation^[^ ^43^, ^44^ ^]^

(1) $$V_{\mathrm{OC}}=E_{a}/q\;-AkT/qln\left(J_{00}/J_{L}\right)$$

Where *E*_a_ is a recombination activation energy, and the *J*_00_ is a prefactor dependent on recombination paths, *A* is an ideality factor, and *k* is Boltzmann constant. According to the equation, *E*_a_ can be obtained by the linear extrapolation of high temperature *V*_OC_ data to 0 k, where A and *J*_00_ are not significantly temperature dependent. *E*_a_ should equal the bandgap energy of Sb_2_Se_3_ absorber in the case of the bulk recombination, whereas it is lower than the bandgap in case of interface recombination.^[^ ^43^, ^45^, ^46^ ^]^ As shown in Figure 6h, the *E*_a_ of Sb_2_Se_3_ solar cells with CdS single buffer was only 0.804 eV, much smaller that the bandgap of the Sb_2_Se_3_ (≈1.24 eV, derived from EQE measurement) absorber layer. On the other hand, all the devices with double buffers exhibit higher *E*_a_ values (C11 0.926, C9 0.951, C7 1.06, C5 0.878 eV) than the cell with single CdS buffer. This behavior of *E*_a_ could be interpreted from the perspective of band structure of the heterojunction interface. The CBM of CdS is lower than that of Sb_2_Se_3_ absorber in the CdS/Sb_2_Se_3_ heterojunction, which enhances the possible interface recombination due to cross‐recombination.^[^ ^47^ ^]^ In this case, the activation energy *E*_a_ is smaller than the bandgap of Sb_2_Se_3_ absorber, and may be close to the energy difference between the CBM of CdS and the VBM of Sb_2_Se_3_. This results in a lower *V*_OC_. The negative CBO (cliff‐like) became positive (spike‐like) after the introduction of In_2_S_3_‐CdS composite buffers, and the cross‐recombination paths are reduced and the interface recombination rate decreases, which would lead to an increase in *E*_a_ and be more beneficial to achieve higher *V*_OC_.^[^ ^48^, ^49^ ^]^ Moreover, If the CBO becomes too large, the photogenerated electrons transferring from the Sb_2_Se_3_ absorber toward the top TCO contact are blocked by the high electron barrier at the junction interface, resulting in a decrease in photoresponse.

## 3 Conclusions

In conclusion, a low‐temperature solution‐processable In_2_S_3_ nanoflake/CdS composite layer is an efficient buffer layer for the Sb_2_Se_3_ nanorod array solar cells. Morphological and structural analyses revealed that In_2_S_3_‐CdS composite shell compactly covers the surface of Sb_2_Se_3_ nanorod. The best‐performing device based on In_2_S_3_‐CdS composite buffers achieved a PCE of 9.19% (*V*_OC_ 461 mV), compared to that of the single In_2_S_3_ nanoflake buffer‐based device of 3.41% (302 mV) and single CdS buffer‐based device of 7.39% (405 mV). The gradient band structure, high‐transmittance at short wavelengths, and low recombination of photogenerated carriers at the Sb_2_Se_3_/buffer nanorod array junction interfaces are responsible for the enhanced efficiency.

## 4 Experimental Section

### Preparation of In_2_S_3_‐CdS Nanocomposite Films

The In_2_S_3_ nanoflake film was grown using thioacetamide (C_2_H_5_NS, 99%, MACKLIN) and indium chloride (InCl_3,_ 99.999%, MACKLIN) as S and In precursors, respectively. In a typical procedure, 1.26 g citric acid (C_6_H_8_O_7,_ AR, 99.5%, MACKLIN) and 0.45 g C_2_H_5_NS were added into 60 mL of 25 × 10^−3^ m InCl_3_ solution with stirring. Then, the precursor solution was transferred to Teflon‐lined autoclave and the prepared Mo/MoSe_2_/Sb_2_Se_3_ substrate was immersed in the reaction solution. The autoclave was put into a preheated box furnace and kept at 80 °C for 100 min. After the reaction, the samples were washed with deionized (DI) water and ethanol and dried at 100 °C in the air for 30 min. Finally, the CdS layer was prepared by a typical CBD method. Add 15 × 10^−3^ m of cadmium sulfate (CdSO_4_, AR, 99%, MACKLIN) and 37 × 10^−3^ m of thiourea (AR, 99%, MACKLIN) and 58.9 mL of ammonia (28%) to 585 mL of deionized water, stirring in a water bath (70 °C) for different time (5, 7, 9, 11, and 13 min), and the samples were washed with DI water and dried with clean dry air.

### Fabrication of Sb_2_Se_3_ Nanorod Array Solar Cells

Sb_2_Se_3_ nanorod array solar cells were fabricated in a substrate configuration (glass/Mo/MoSe_2_/Sb_2_Se_3_/buffer layer/i‐ZnO/AZO), as described in our previous work.^[^ ^25^ ^]^ The Mo back contact layer was prepared by 1200 W DC sputtering under a 0.3 Pa argon, in which the obtained Mo thickness was about 800 nm. Then, the as deposited W layer was transferred into a vacuum chamber for selenization. During the selenization, the vacuum chamber pressure was pumped down to 10^−4^ Pa and the substrate temperature was kept at 620 °C for 30 min. Approximately 1 µm thick Sb_2_Se_3_ nanorod array absorber layers were deposited by close space sublimation by a process of our previous recipe:^[^ ^25^ ^]^ the deposition started when the pressure was below 10^−2^ Pa. First, the source and sample holder were warmed up to 480 and 270 °C, respectively, in 200 s, and maintained at the high temperatures for 100 s to obtain the Sb_2_Se_3_ absorbers with thickness of 1 µm. Different buffers (In_2_S_3_ buffer, In_2_S_3_/CdS composite buffers, and CdS buffer) were prepared by the above process. Window layers of i‐ZnO and AZO were deposited by RF magnetron sputtering to a thickness of 70 and 300 nm, respectively.

### Characterization and Measurement

The morphology of the films was observed by high resolution field emission SEM (FEI nova nano SEM450) and atomic force microscopy (Veeco Multimode 8). XPS and UPS measurements were carried out using an X‐ray photoelectron spectrometer and an ultraviolet photoemission spectrometer (ESCALAB 250Xi, Thermo Scientific), respectively. For the In‐depth XPS and UPS measurement, an Ar^+^ ion source with energy of 2 KeV was used, the reference etching rate was 0.60 nm s^−1^ for Ta_2_O_5_. The optical properties of the thin film were measured using a spectrophotometer equipped with an integrating sphere (Perkin‐Elmer Lambda 950). To characterize the device performance, J‐V measurements were performed on the devices using an AM1.5 solar simulator equipped with a 300 W xenon lamp (Model No. XES‐100S1, SAN‐EI, Japan). The external quantum efficiency (EQE) was measured using an Enlitech QER3011 system (Enlitech) equipped with a 150 W xenon light source. The photocurrent decay under short circuit and photovoltage decay under open‐circuit conditions were measured by an electrochemical workstation (ZAHNER GIMPS, Germany).

## Conflict of Interest

The authors declare no conflict of interest.

## Supporting information

Supporting Information

## Data Availability

Research data are not shared.
