# Supplementary material for: Conduction Band Energy‐Level Engineering for Improving Open‐Circuit Voltage in Antimony Selenide Nanorod Array Solar Cells
Source: Adv Sci (Weinh). 2021 Jun 10;8(16):2100868. doi: 10.1002/advs.202100868 (PMC8373166; doi:10.1002/advs.202100868)
Supplement: Supplementary file 1 — Supporting Information [file ADVS-8-2100868-s001.pdf]

WILEY-VCH

© 2021 Wiley-VCH GmbH

ADVANCED SCIENCE
Open Access

Supporting Information

for *Adv. Sci.*, DOI: 10.1002/advs.202100868

Conduction Band Energy-level Engineering for Improving Open-circuit Voltage in Antimony Selenide Nanorod Array Solar Cells

Tao Liu, Xiaoyang Liang, Yufan Liu, Xiaoli Li, Shufang Wang, Yaohua Mai, and *Zhiqiang Li**

Supporting Information

Conduction Band Energy-level Engineering for Improving Open-circuit Voltage in Antimony Selenide Nanorod Array Solar Cells

*Tao Liu, Xiaoyang Liang, Yufan Liu, Xiaoli Li, Shufang Wang, Yaohua Mai, Zhiqiang Li**

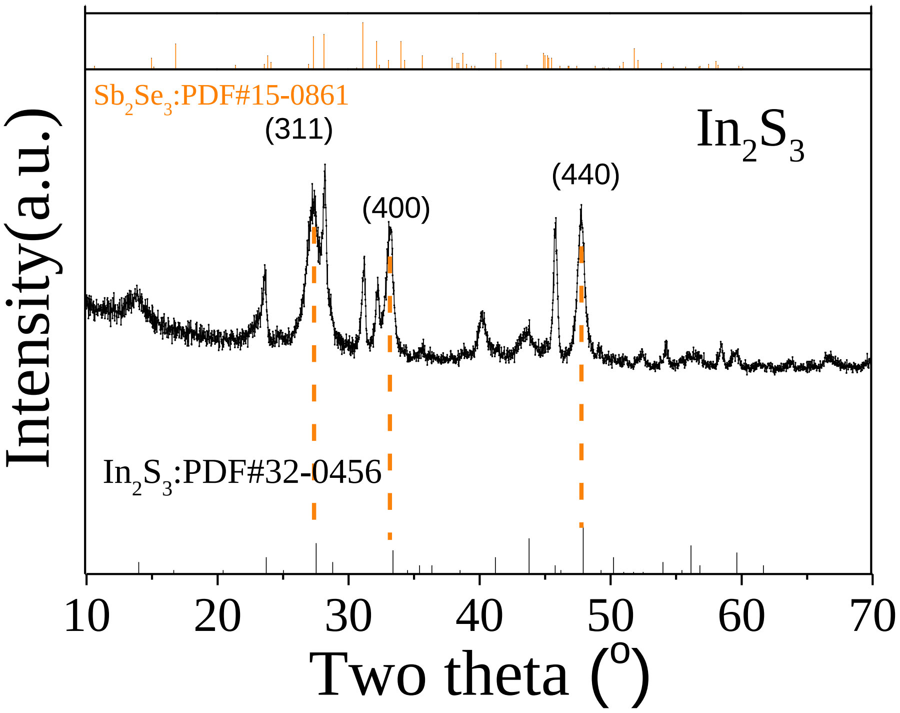

Figure S1 XRD pattern of the solution-processed In_2S_3 nanosheet layer.

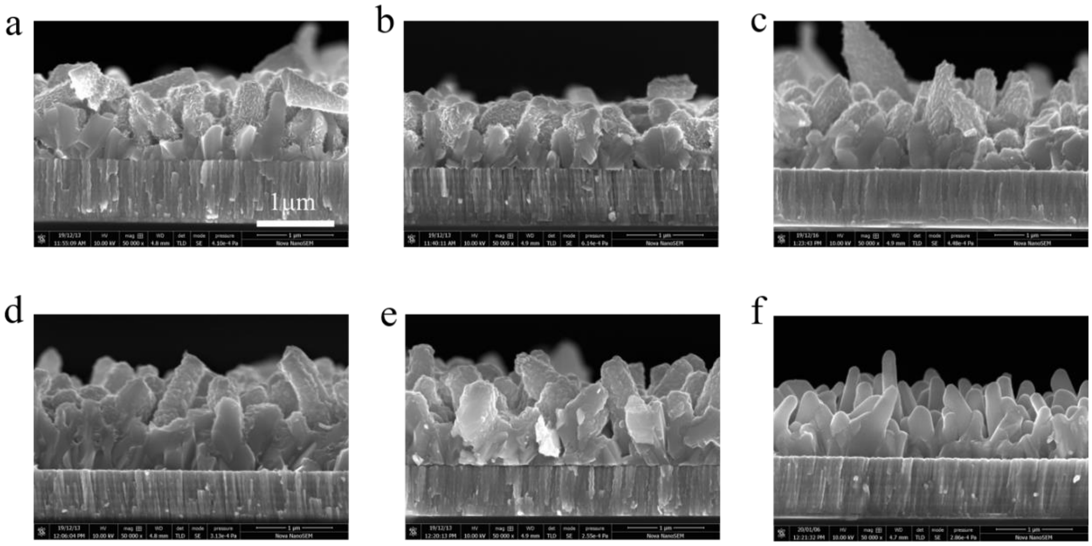

Figure S2 Cross section SEM images of Sb_2Se_3 nanorod arrays with different buffer layers. a, single In_2S_3, b-e, In_2S_3-CdS composites (b, C5; c, C7; d, C9; e, C11), and f, single CdS.

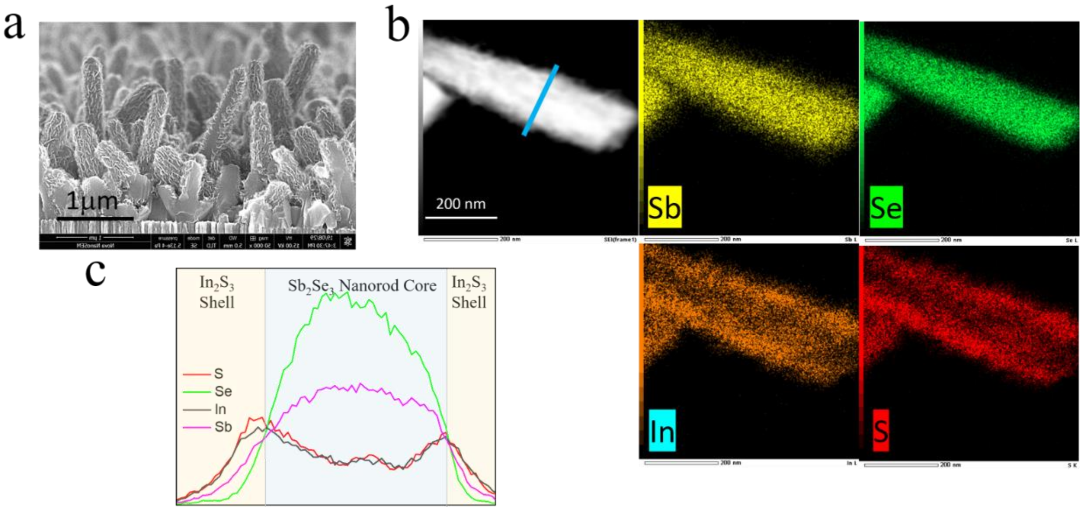

Figure S3 SEM image, EDX mapping and line scan of the Sb_2Se_3/In_2S_3 core/shell nanorod.

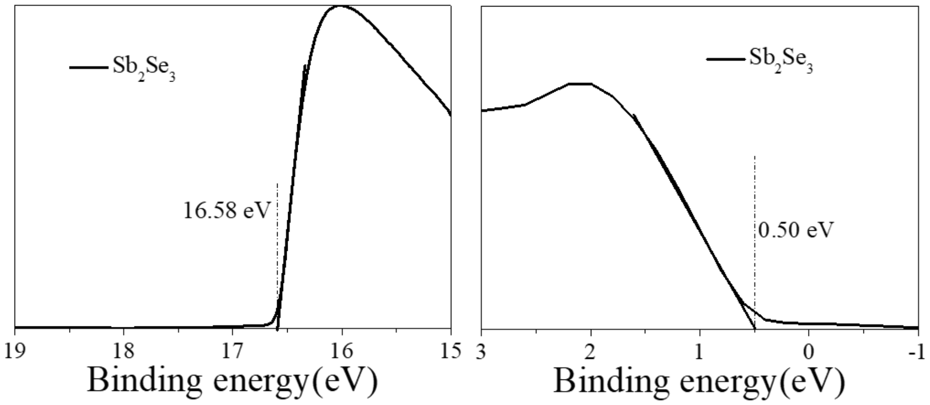

Figure S4 UPS curves of Sb_2Se_3 nanorod arrays. Fermi energy was obtained as 4.64 eV by subtracting the spectrum onset of 16.58 eV with the UP energy of 21.22 eV. The distance between the VBM and the Fermi level was about 0.50 eV. The bandgap of Sb_2Se_3, obtained from the differentiation of the EQE spectrum, is 1.24 eV. The VBM and CBM of the Sb_2Se_3 nanorod arrays locates at 5.14 eV and 3.90 eV, respectively.

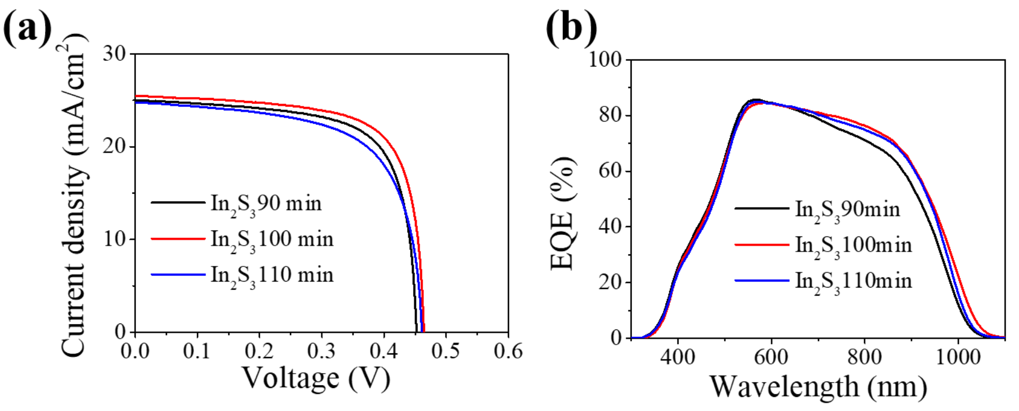

Figure S5 (a) J-V curves and (b) EQE spectra for the Sb_2Se_3 solar cells with different In_2S_3 deposition times.

Table S1 Device performance parameters for the Sb_2Se_3 solar cells with different In_2S_3 deposition times.

Buffer	V_{OC} (mV)	J_{SC} (mA cm^{-2})	FF (%)	PCE (%)
**In_2S_3 90 min**	448±7 (450)	24.1±0.8 (25.0)	67.7±2.8 (65.6)	7.2±0.7 (7.4)
**In_2S_3 100 min**	463±3 (464)	24.8±1.1 (25.4)	69.0±1.8 (67.2)	7.8±0.3 (7.9)
**In_2S_3 110 min**	450±6 (460)	24.3±0.8 (24.7)	67.8±3.7 (67.0)	7.6±0.4 (7.6)

The effect of the thickness of In_2S_3 layers on the current density-voltage (J-V) curves and on the EQE spectra are shown in Figure S5 and Table S1. The effect of In_2S_3 deposition time between 90 and 110 min was slightly to the device performance. The device with In_2S_3-100 min layer exhibited a champion power conversion efficiency of 7.9%, with a short-circuit current (J_{SC}) of 25.4 mA cm^{-2}, an open-circuit voltage (V_{OC}) of 464 mV, and a fill factor (FF) of 67.2%. As shown in Figure R4b, all the devices exhibited similar behaviors in the short wavelength range, while the cells with In_2S_3-100 min and In_2S_3-110 min layers displayed slightly higher EQE values than the In_2S_3-90 min device.

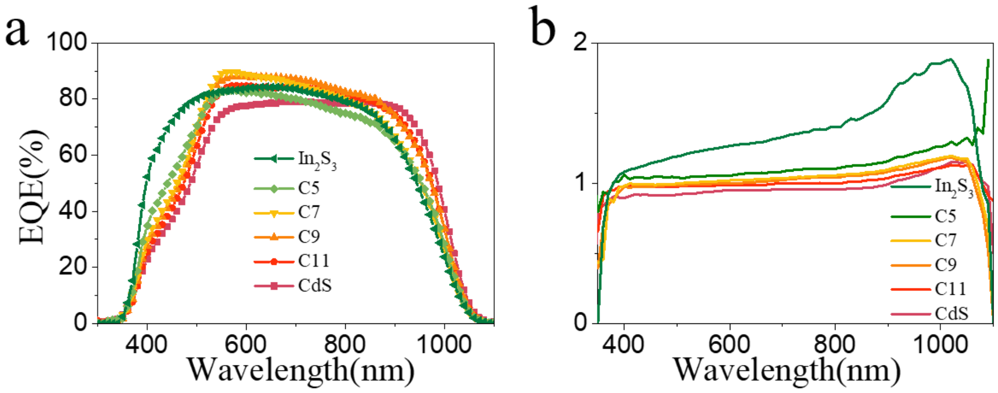

Figure S6 a, EQE response measured at -0.5V and b, EQE(-0.5V)/EQE(0V) ratios of the Sb_2Se_3 nanorod array solar cells.
